# Changes in research competency of public health personnel following an empowerment-based capacity development program in Thailand: a convergent parallel mixed-methods study

**DOI:** 10.3389/fpubh.2026.1950760

**Published:** 2026-09-23

**Authors:** Kittiporn Nawsuwan, Noppcha Singweratham, Natthawut Suriya, Navaporn Damsangsawat, Ratdawan Klungklang

**Affiliations:** 1 Boromarajonani College of Nursing, Faculty of Nursing, Praboromarajchanok Institute, Songkhla, Thailand; 2 Faculty of Public Health, Chiang Mai University, Chiang Mai, Thailand; 3 Srimahasarakham Nursing College, Faculty of Nursing, Praboromarajchanok Institute, Maha Sarakham, Thailand; 4 Boromarajonani College of Nursing, Faculty of Nursing, Praboromarajchanok Institute, Khon Kaen, Thailand

**Keywords:** capacity development, empowerment, feasibility health study, mixed-methods research, program evaluation, public health workforce, research competency, systems strengthening

## Abstract

**Introduction:**

Research competency among frontline public health personnel is a prerequisite for evidence-informed local health systems, yet most capacity-building programs in low- and middle-income settings are short didactic courses that are rarely evaluated with both outcome and process data. This study examined changes in self-reported research competency among public health personnel who completed an Empowerment-Based Capacity Development Program designed to convert routine public health work into publishable research, and explored how participants themselves accounted for those changes. The quantitative outcome sits at Kirkpatrick level 2 (self-reported learning) and the qualitative strand adds level 1 (reaction); neither strand reaches level 3 (behavior) or level 4 (results). The evaluation is presented as preliminary implementation and feasibility evidence rather than as an effectiveness trial.

**Methods:**

A convergent parallel mixed-methods design was used. The quantitative strand was a single-group pretest–posttest study without a comparison group, conducted with 30 purposively sampled public health personnel from three self-selected service settings in Nakhon Si Thammarat, Surat Thani, and Khon Kaen provinces, Thailand. Participants completed a five-session, six-component empowerment program (policy, preparation, practice, publishing, positive reinforcement, and passion) delivered over one academic year. Self-reported research competency was measured with an adapted 47-item research competency questionnaire; the factor structure of the original instrument was reported previously, whereas evidence generated for the present study was limited to content validity of the adapted items (item-objective congruence 0.67–1.00) and internal consistency in a separate pilot of 30 personnel (Cronbach's α = 0.84). Data were analyzed with paired *t*-tests and Wilcoxon signed-rank tests with effect sizes. The qualitative strand comprised three focus group discussions (*n* = 30) held after the program and analyzed by content analysis. Strands were merged in a joint display with pre-specified mapping criteria and interpreted through an explicit meta-inference.

**Results:**

Self-reported overall research competency was higher at posttest than at pretest, rising from 3.13 (SD = 0.27) to 4.27 (SD = 0.35) out of 5 (within-participant mean difference 1.14, 95% CI 0.98–1.30; *p* < 0.001; Cohen's dz = 2.58), with increases in all eight domains (dz = 1.04–1.81; Wilcoxon *r* = 0.59–0.60). Content analysis yielded nine categories grouped into four themes: crossing the expert barrier; from first publication to research as ordinary work; learning with others and putting findings to use; and the limits of intention workload, dissemination and dependence on scaffolding. Integrating the strands indicated that change concentrated in the motivational and evaluative domains while technical and dissemination competencies lagged, and that participants attributed that lag not to residual knowledge deficits but to service work taking priority and to reliance on mentor scaffolding for the final steps of publication. At the structural level, construct-level consistency was found for five of the eight domains, illustration or expansion for three, and two participant-reported outcomes interprofessional exchange and the reframing of research as routine work were absent from the instrument; no discordance was identified.

**Discussion:**

Participation in an empowerment-based, practice-embedded program was associated with large within-participant increases in self-reported research competency, and participants' retrospective accounts described the change as a shift from research as an expert activity to research as an extension of routine work that nonetheless stops short of independent dissemination. Because the design was single-group and both strands relied on self-report collected by the team that delivered the program, these findings constitute feasibility and acceptability evidence rather than evidence of causal effectiveness. Controlled designs with longer follow-up and objective research-productivity indicators are required before wider scale-up, and competency instruments in this field require items covering collaboration and the integration of research into routine work.

## Introduction

Health systems worldwide are confronting a converging set of pressures: pandemics and emerging infectious diseases, antimicrobial resistance, the rising burden of noncommunicable diseases, and environmental health hazards (1, 2). Meeting these pressures requires a workforce that can define local problems, generate local evidence, and act on it. The Ottawa Charter framework for health promotion identifies the development of personal skills and the reorientation of health services as two of its five strategic actions (3), both of which depend on the analytic capacity of frontline personnel rather than on centrally produced evidence alone.

Thailand is widely cited as an example of an equitable health system in a middle-income country, built on primary health care, universal health coverage, and regional field epidemiology training networks (4). Much of this achievement is attributed to the competency of personnel working under the Ministry of Public Health. Consistent with this, the People Excellence pillar of Thailand's 20-Year National Public Health Strategy (2017–2036) explicitly requires continuous learning and research engagement across the workforce (5), echoing international frameworks in which research and evaluation are treated as essential public health services (6).

In practice, however, frontline personnel in Thailand and comparable settings publish little. Documented barriers include heavy service workloads, limited protected time, scarce methodological support, weak access to literature, and low confidence in statistics (7). A mixed-methods study of health providers in Tanzania found the same pattern and identified motivation to solve community problems, opportunities for professional growth, and collaborative structures as the main enablers of research participation (8). A recent concept analysis mapped the attributes, antecedents and consequences of research capacity strengthening for health professionals in low- and middle-income countries, and showed how heterogeneously the concept is defined and operationalized across this literature (9).

A systematic meta-narrative review of the same field reached a more specific conclusion: fragmented, project-based training has yielded limited and poorly documented gains, because power relations, institutional systems, and the links between research, policy and practice rather than knowledge transfer alone determine whether research activity is sustained (10). Frameworks developed for planning and evaluating research capacity building make the same point structurally, specifying skills and confidence, partnerships, closeness to practice, dissemination, infrastructure, and continuity as six distinct principles that a single training event cannot address (11).

Empowerment theory offers a mechanism for addressing precisely those non-cognitive barriers. Empowerment is understood as a social process through which individuals recognize their own capabilities, gain control over decisions affecting their work, and act on that control (12, 13). Applied to research capacity, it reframes the learner from a recipient of methods instruction into an agent who identifies a local problem and carries it through to dissemination. Building on this premise, Nawsuwan et al. (14) proposed a six-component empowerment-based model for producing academic work among village health volunteers and health officials, comprising policy, preparation, practice, publishing, positive reinforcement, and passion. Research competency itself is multidimensional, spanning knowledge, methodological skill, supportive personal traits, attitude, motivation, valuation of research, application of findings, and dissemination (15).

Structured research training and mentorship programs are not new to low- and middle-income settings, and several established programs are considerably more intensive than the one evaluated here. A year-long Intermediate Operational Research Training course in Rwanda combined classroom teaching with sustained individual mentorship; 53 of 55 selected trainees completed it, they co-first-authored 28 manuscripts of which 27 were published in peer-reviewed journals, and the median cost was US$908 per trainee (16). Research capacity building integrated into five Population Health Implementation and Training partnerships across sub-Saharan Africa delivered 54 research trainings, 56 mentor–mentee relationships and 116 manuscripts over 5 years, alongside investment in research administration and infrastructure (17). In Tanzania, the Community of Young Research Peers combined vertical mentorship by senior researchers with horizontal peer-to-peer mentorship among master's-level early-career researchers, and reported publications, grant applications and onward mentorship of undergraduates (18). These programs show that mentorship-rich models can generate documented research outputs. They have generally been delivered to selected early-career researchers or academic staff, supported by dedicated external or partnership funding, and evaluated primarily through counts of outputs. The present study does not claim to introduce a new or more comprehensive model, and we explicitly do not position the program as novel in its components. Its contribution is narrower and concerns a different delivery constraint: what happens when a mentorship-supported research cycle is compressed into 10 facilitated days across one academic year and offered to district-level personnel who remain in full-time service roles, and how those personnel themselves account for what changed.

Three gaps motivated this study. First, evaluations of research capacity building remain dominated by outcomes collected at the level of participant reaction and self-rated learning immediately after training, and the instruments available for assessing capacity development more comprehensively are unevenly applied (10, 11). Interpreting such evaluations requires being explicit about which level of educational outcome has actually been assessed. In Kirkpatrick's widely used four-level scheme this is the distinction between reaction (level 1) and learning (level 2) on the one hand, and behavior in the workplace (level 3) and organizational results (level 4) on the other (19); the levels are best used descriptively, to state what a given design can support, rather than as a quality hierarchy (20). Second, quantitative evaluations seldom explain why participants believe a program worked, whereas qualitative studies rarely quantify how much self-reported competency changed; few studies bring both to bear on the same program (21). Third, empowerment-based models have been tested mainly with teachers and university lecturers (22, 23) rather than with district-level public health personnel who carry routine service responsibilities.

This study therefore had two aims, both deliberately specified in non-causal terms. The primary aim was to assess within-participant change in self-reported research competency, overall and across eight domains, between the beginning and the end of an Empowerment-Based Capacity Development Program. The secondary aim was to explore how participants perceived and explained that change. Where the word “effectiveness” is used anywhere in this article in relation to the program, it should be read as within-person pre–post change under routine implementation conditions, and not as a causal effect attributable to the program. The model adapts Gibson's four-stage empowerment process discovering reality, critical reflection, taking charge, and holding on (12, 13) to the six programmatic components described above; the conceptual framework is shown in Figure 1. We anticipated that self-reported research competency would be higher at posttest than at pretest, overall and in each of the eight domains, and we used a convergent parallel design so that measured change and participant accounts could be interpreted together. Because the qualitative strand was collected only after the program, participants' accounts are treated throughout as retrospective perceptions of change rather than as independent verification that the measured change occurred.

**Figure 1:**
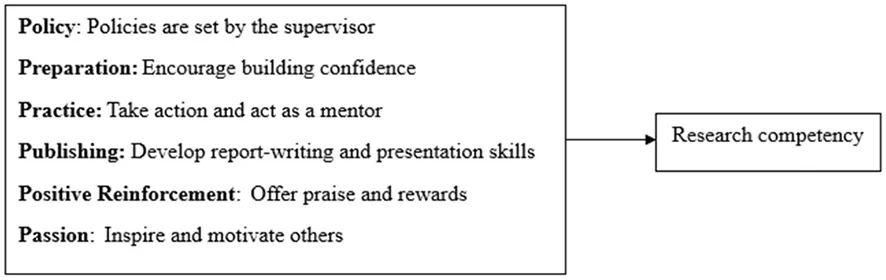
Conceptual framework.

## Materials and methods

### Study design and reporting

We used a convergent parallel mixed-methods design (24, 25), in which quantitative and qualitative data were collected during the same period, analyzed independently, and merged at the interpretation stage. The quantitative strand was a single-group pretest–posttest study; the qualitative strand used focus group discussions. Equal priority was given to both strands (QUAN + QUAL). In educational-evaluation terms, the outcomes assessed correspond to Kirkpatrick levels 1 and 2: the quantitative outcome is self-reported learning (level 2), and the focus groups additionally capture participants' reactions to the program (level 1). Neither strand reaches level 3 (observed change in workplace behavior) or level 4 (organizational results such as completed protocols, publications, or service change) (19). We use this scheme descriptively, to make explicit what our measures can and cannot support, while recognizing that the levels are not a hierarchy of study quality and that their assumptions fit relatively simple instructional designs (20). Reporting follows the Good Reporting of a Mixed Methods Study (GRAMMS) criteria (21) for the study as a whole and the Consolidated Criteria for Reporting Qualitative Research (COREQ) (26) for the qualitative strand; completed checklists are provided as Supplementary material.

### Rationale for the single-group design and threats to internal validity

A controlled design was considered during planning and judged not feasible in the participating settings, for three reasons. First, the program required the written commitment of the head of each organization to release staff for 10 working days spread across one academic year; no participating organization was willing to release additional staff solely to serve as an untreated comparison group, and administrators regarded random assignment of individual staff within small district teams as inequitable, because the program was viewed locally as a professional development opportunity. Second, the eligible workforce at each site was small (14, 18 and 12 staff met the inclusion criteria at the district public health office, the hospital and the primary healthcare unit respectively), so contamination between arms within a site was likely: staff share offices, caseloads, meetings and supervision, and the program required participants to present their work publicly to colleagues. Third, the program was commissioned and scheduled as a service-development activity rather than as a trial, and the study was designed to evaluate it as implemented in routine conditions. The consequence is explicit: this design supports the description of within-participant change and the assessment of feasibility and acceptability, but it cannot rule out maturation, history, testing and re-testing effects, regression to the mean, or concurrent organizational initiatives as alternative explanations for the change observed. Three safeguards were used to limit, though not to eliminate, these threats. Pretest and posttest questionnaires were administered by research assistants who took no part in delivering the program. Responses were anonymous and linked across time points only by a participant-generated code, so that neither facilitators nor supervisors could attribute any response to an individual. Finally, at the final session the head of each organization confirmed in writing that no other research training, research mentorship or academic-writing activity had been provided to participants during the study period. No adjustment can compensate for the absence of a comparison group, and the findings are reported accordingly throughout.

### Setting and site selection

An open call for participation was circulated to public health organizations under the Ministry of Public Health; participation was therefore voluntary at the organizational level, and the participating sites were self-selected. 10 organizations expressed interest and were assessed by the research team against three readiness criteria: written commitment from the head of the organization, availability of at least 10 eligible personnel, and willingness to release staff for all program sessions. Three organizations were purposively selected: Ron Phibun District Public Health Office (Nakhon Si Thammarat province), Phunphin Hospital (Surat Thani province), and a primary healthcare unit in Khon Kaen province. The three sites represent district administration, hospital, and primary care settings respectively, and were chosen to maximize contextual variation. Because selection was conditional on demonstrated organizational readiness and on a prior commitment to protect staff time, the participating sites should be understood as favorable-case settings rather than as typical district organizations, and this is taken into account when the findings are interpreted.

### Participants and sampling

Participants were public health personnel employed at the three selected sites. Purposive sampling was used with the following inclusion criteria: (i) completion of at least upper secondary education (Grade 12 or equivalent); (ii) basic computer literacy; and (iii) a written commitment to complete the full program. Personnel who were on long-term leave or scheduled for transfer during the study period were excluded. 10 participants were recruited per site (*n* = 30). No participant withdrew, and complete pretest and posttest data were available for all 30 participants. Participants were thus both purposively selected and self-selected: they volunteered, obtained supervisory release, and committed in writing in advance to attend every session. Together with the organizational selection described above, this means that the sample combines organizational readiness with unusually high individual motivation. Such selection is likely to have produced more favorable outcomes than would be expected if the same program were delivered to unselected personnel, and the 100% retention rate should be read as a property of this highly selected group rather than as a general indicator of the program's feasibility in routine conditions.

### Sample size

Sample size was estimated in G^*^Power 3.1 (27) for a two-tailed paired-samples *t*-test (means: difference between two dependent means). A previous evaluation of an empowerment-based model in a comparable population reported an effect size of 1.69 (14), which would have required only six participants and was judged implausibly large for planning purposes. We therefore adopted a conservative medium effect size of *d* = 0.50 (28) with α = 0.05 and power = 0.95, yielding 27 participants. Allowing for approximately 10% attrition, the target was set at 30 participants (10 per site). This calculation determines the precision with which within-participant change can be estimated; it does not address the design's inability to attribute that change to the program.

### Intervention: the empowerment-based capacity development program

The program operationalized Gibson's empowerment process (12, 13) through six components — policy, preparation, practice, publishing, positive reinforcement, and passion (14) — delivered as five facilitated sessions over one academic year, each lasting two consecutive days, hosted in rotation across the three sites. Each session day ran from 09:00 to 16:00 with a one-hour break, giving 6 h of scheduled activity, so the program comprised 10 program days and 60 h of face-to-face contact in total. All 30 participants attended each session together. Three doctorally prepared public health researchers with prior experience of mentoring practitioner research facilitated every session, giving an overall facilitator-to-participant ratio of 1:10; each facilitator acted as the designated mentor for the 10 participants from one site. Every session day combined the same four elements in the same order: group discussion of local service problems (90 min), a short methodological lecture (60 min), supervised writing practice on the participant's own project (120 min), and public presentation of progress with structured peer and facilitator feedback (90 min), so that each participant advanced one project of their own from question to manuscript. Between sessions, participants worked on their projects in their own workplaces, with an expected workload of approximately 4 h per month, and a median of 3.5 h per month reported in the participant logbooks, and received asynchronous mentoring by e-mail and LINE messaging in individual and site-group chats, with facilitators responding within two working days; across the year the three site-group chats recorded a median of 11 mentoring exchanges per participant. Materials provided to every participant were a 48-page program handbook, Word templates for the protocol, the data collection form and the manuscript, a one-page checklist for each session deliverable, and a reading list of eight exemplar practitioner studies published in Thai journals. Each session had a defined deliverable that participants submitted before the following session: a research question and problem statement (session 1); a full protocol with instrument and sample size (session 2); a data collection and record-keeping plan with preliminary data (session 3); an analysis and results section (session 4); and a complete manuscript including a discussion section (session 5). The structure, contact time, between-session mentoring, and expected participant output of each session are summarized in Table 1.

**Table 1:** Structure, contact time, between-session mentoring, and expected participant output of the five program sessions.

Session (2 days each)	Core activities and contact time	Between-session mentoring and workload	Participant deliverable before next session	Empowerment stage
1	Group discussion of problems and needs in academic work production and sharing of previous experience (90 min); lecture on topic selection and searching credible databases (60 min); supervised writing practice (120 min); group presentation and summary of learning (90 min). Same four-element structure and timings on every session day; 6 h per day, 12 h per session	E-mail and LINE individual and site-group chats; facilitator response within 2 working days; expected participant workload ≈4 h/month	Research question and problem statement	Discovering reality
2	Presentation of candidate research topics and supporting evidence; lecture on research design, sample size, instrument development, instrument quality, and continuous quality improvement; supervised writing practice; progress presentation; group summary	As above; individual review of draft protocols by the site mentor	Full protocol with instrument and sample size calculation	Critical reflection
3	Presentation of progress; lecture on data collection and record keeping; supervised writing practice; progress presentation; group summary	As above; troubleshooting of field data collection	Data collection and record-keeping plan with preliminary data	Taking charge
4	Presentation of progress; lecture on data analysis and presentation of results; second plan–do–check–act cycle; supervised writing practice; progress presentation; group summary	As above; analysis support and review of results tables	Analysis and results section	Taking charge
5	Presentation of progress; lecture on discussion writing and academic dissemination; supervised writing practice; oral presentation of completed work; group discussion of key success factors	As above; manuscript feedback	Complete manuscript including discussion	Holding on

Each session lasted two consecutive days, giving 10 program days and 60 h of face-to-face contact in total. All sessions were delivered face to face and facilitated by the same three doctorally prepared researchers (facilitator-to-participant ratio 1:10), each acting as designated mentor for the 10 participants of one site. LINE is a messaging application in general use in Thailand.

### Measures and their psychometric basis

Self-reported research competency was measured with a questionnaire adapted from Semrum et al. (15), developed for public health personnel in subdistrict health-promoting hospitals. Part 1 records seven characteristics of the respondent. Part 2 contains 46 items across eight domains: knowledge of research development (7 items), ability to conduct research (9 items), research-supportive personal traits (7 items), attitude toward research development (4 items), motivation for research development (4 items), appreciation of the value of research (4 items), ability to apply research findings to work improvement (6 items), and ability to present academic work (5 items). Items are rated on a five-point scale from 1 (strongly disagree / least frequent practice) to 5 (strongly agree / most frequent practice), and domain scores are the mean of their items. All items are self-report; no observed or objectively assessed measure of competence was collected.

The instrument is described here as an adapted 46-item research competency questionnaire with previously reported validity evidence, rather than as a fully validated scale, and the sources of that evidence are distinguished as follows. The item pool and the eight-domain structure derive from the original instrument, whose factor structure was established by Semrum et al. (15) in a separate sample of public health personnel; that evidence belongs to the original instrument, not to the present study. Evidence generated for the present study is limited to two elements. First, content validity of the adapted items was assessed by three experts (two public health academics and one research methodologist), yielding an item-objective congruence index of 0.67 to 1.00. Second, internal consistency was examined in a pilot with 30 public health personnel in Songkhla province who did not take part in the main study, where Cronbach's α was 0.84 for the total scale (domain values 0.71–0.89). A pilot of this size supports a preliminary judgement of internal consistency only, and does not constitute comprehensive validation. In the main study sample, Cronbach's α for the total scale was 0.87 at pretest and 0.91 at posttest (domain values 0.72–0.88 and 0.76–0.90, respectively; the lowest value at both time points was for the four-item attitude domain). No confirmatory factor analysis or other construct-validity analysis was conducted in the present sample, which is far too small for such analysis given 46 items and 30 respondents. The eight domain scores are therefore reported as descriptive subscales with acceptable internal consistency, not as psychometrically confirmed constructs in this population.

For the qualitative strand, a semi-structured focus group guide was developed by the research team from the eight competency domains and reviewed by the same three experts. It opened with a single broad question inviting participants to describe how their ability to produce academic work had changed, followed by non-directive probes.

### Quantitative data collection

After ethical approval and informed consent, research assistants who were not involved in delivering the program administered the questionnaire immediately before the first session (pretest) and within 2 weeks after the final session (posttest). Questionnaires were completed anonymously and linked across time points by a participant-generated code, which was known only to the participant and was not recorded against any identifying information.

### Qualitative data collection

Three focus group discussions, one per site, were held within 2 weeks after the final session; all qualitative data therefore describe the change retrospectively, as participants recalled and interpreted it at the end of the program, and no qualitative data were collected at baseline. Each group comprised the 10 participants from that site; no non-participants, supervisors or managers were present. Discussions were moderated by the first author, with two trained note-takers, lasted 90–120 min, and were audio-recorded with written permission. At the end of each session the moderator summarized the main points and invited participants to correct them (member checking). Recordings were transcribed verbatim in Thai; quotations reported here were translated into English by a bilingual researcher and back-translated for accuracy. Participants are identified by number only.

### Data analysis

Quantitative data were analyzed in SPSS version 26. Participant characteristics and domain scores were summarized as frequencies, percentages, means, standard deviations, and ranges. Normality of the pretest–posttest difference scores was examined with the Shapiro–Wilk test. Differences were normally distributed for six domains and for the overall score (*p* = 0.089–0.562) and were analyzed with paired *t*-tests; the distributions for research-supportive traits and attitude toward research departed from normality (*p* = 0.035 and 0.017) and were analyzed with Wilcoxon signed-rank tests (29). All tests were two-tailed with α = 0.05. Effect sizes are reported as Cohen's dz for paired *t*-tests and as *r* = Z/√N for Wilcoxon tests, with 95% confidence intervals for mean differences. Because the eight domain comparisons were pre-specified and the study is descriptive rather than confirmatory, *p*-values are reported without adjustment for multiplicity and should be read as descriptive.

Qualitative data were analyzed by content analysis following Miller and Crabtree (30): organizing the transcripts, coding, grouping codes into categories, developing themes, examining relationships between themes, and verifying the interpretation. Two researchers coded the transcripts independently; disagreements were resolved by discussion with a third researcher. The nine categories that emerged were subsequently grouped into four themes by consensus among three researchers, on the basis of conceptual proximity rather than frequency, and the grouping was checked against the transcripts before it was accepted. Accounts that described difficulty, doubt or unmet expectation were coded and retained in the same way as accounts of change, and are reported within the themes rather than separately.

### Integration of the two strands

The two strands were merged after separate analysis using a joint display (31). Because the credibility of a joint display rests on how the mapping was made, the procedure and its criteria are stated explicitly. Mapping proceeded in three steps. First, each of the nine qualitative categories was matched to the competency domain whose item content it most closely described; two researchers did this independently, using the 47 item stems rather than the domain labels as the criterion, and disagreements were resolved by discussion. Second, the direction and magnitude of quantitative change in the matched domain was placed alongside the category. Third, the relationship was classified as confirmation (the qualitative category describes the same construct as the domain and points in the same direction), illustration/expansion (the category is related to but not equivalent to the domain, and elaborates, exemplifies or extends it rather than corroborating it), or discordance (the two point in opposite directions). Where a category could plausibly be mapped to more than one domain, or where the correspondence was thematic rather than construct-level, we classified the relationship as illustration/expansion rather than confirmation; this rule was applied deliberately conservatively. One boundary condition applies to the whole display: because qualitative data were collected only after the program, no qualitative category can independently confirm a pre–post change. “Confirmation” in Table 2 therefore denotes construct-level consistency between the two strands at the end of the program, not verification that the measured change occurred or that it was produced by the program. The design and the integration points are shown in Figure 2.

**Table 2:** Joint display integrating the quantitative and qualitative strands, with the basis for each mapping and its classification.

Competency domain (QUAN)	Direction and size of within-participant change	Corresponding qualitative category (QUAL)	Basis of the mapping	Fit
Knowledge of research development	↑ dz = 1.15	Acquiring research knowledge (Theme 1)	Category describes the same construct as the domain items (understanding of the research process and its steps)	Confirmation
Ability to conduct research	↑ dz = 1.81	Thinking systematically; searching for evidence; gaining confidence in data analysis (all Theme 1)	Three categories map onto distinct item stems within the domain (design logic, literature searching, analysis)	Confirmation
Research-supportive personal traits	↑ r = 0.60	Intending to continue (Theme 4)	Category describes intention and self-efficacy, which are related to but not equivalent to the trait items (perseverance, curiosity, attention to detail)	Illustration/expansion
Attitude toward research development	↑*r* = 0.59	Perceiving research as feasible, attitudinal facet (Theme 2)	This category has two facets. Its attitudinal facet — that research is approachable rather than daunting — is an evaluative orientation toward research and matches the attitude item stems; only this facet is mapped here	Confirmation
Motivation for research development	↑ dz = 1.81	Publishing for the first time (Theme 2)	First publication is an experience participants described as motivating, not a measure of motivation; the correspondence is thematic rather than construct-level	Illustration/expansion
Appreciation of the value of research	↑ dz = 1.62	Applying findings to practice (Theme 3)	Category describes use of findings rather than valuation of research; the two constructs are adjacent but distinct	Illustration/expansion
Application of findings to work improvement	↑ dz = 1.49	Applying findings to practice (Theme 3)	Category describes the same construct as the domain items (use of findings to change service or planning decisions)	Confirmation
Ability to present academic work	↑ dz = 1.04 (smallest increase)	Publishing for the first time (Theme 2); the least elaborated category, and the activity participants identified in Theme 4 as least rehearsed	Category describes dissemination activity matching the domain items; participants identified it as least practiced, consistent with the smallest measured increase	Confirmation
Not represented in the instrument	—	Learning across professions (Theme 3)	Interprofessional and cross-site exchange has no corresponding item in the 47-item scale	Expansion
Not represented in the instrument	—	Perceiving research as feasible, routine-work facet (Theme 2)	The second facet of the same category — that research is an extension of work already being done rather than an additional task — is not an attitude item and has no corresponding item anywhere in the 47-item scale, so it is mapped separately from the attitudinal facet in row 4	Expansion

**Figure 2:**
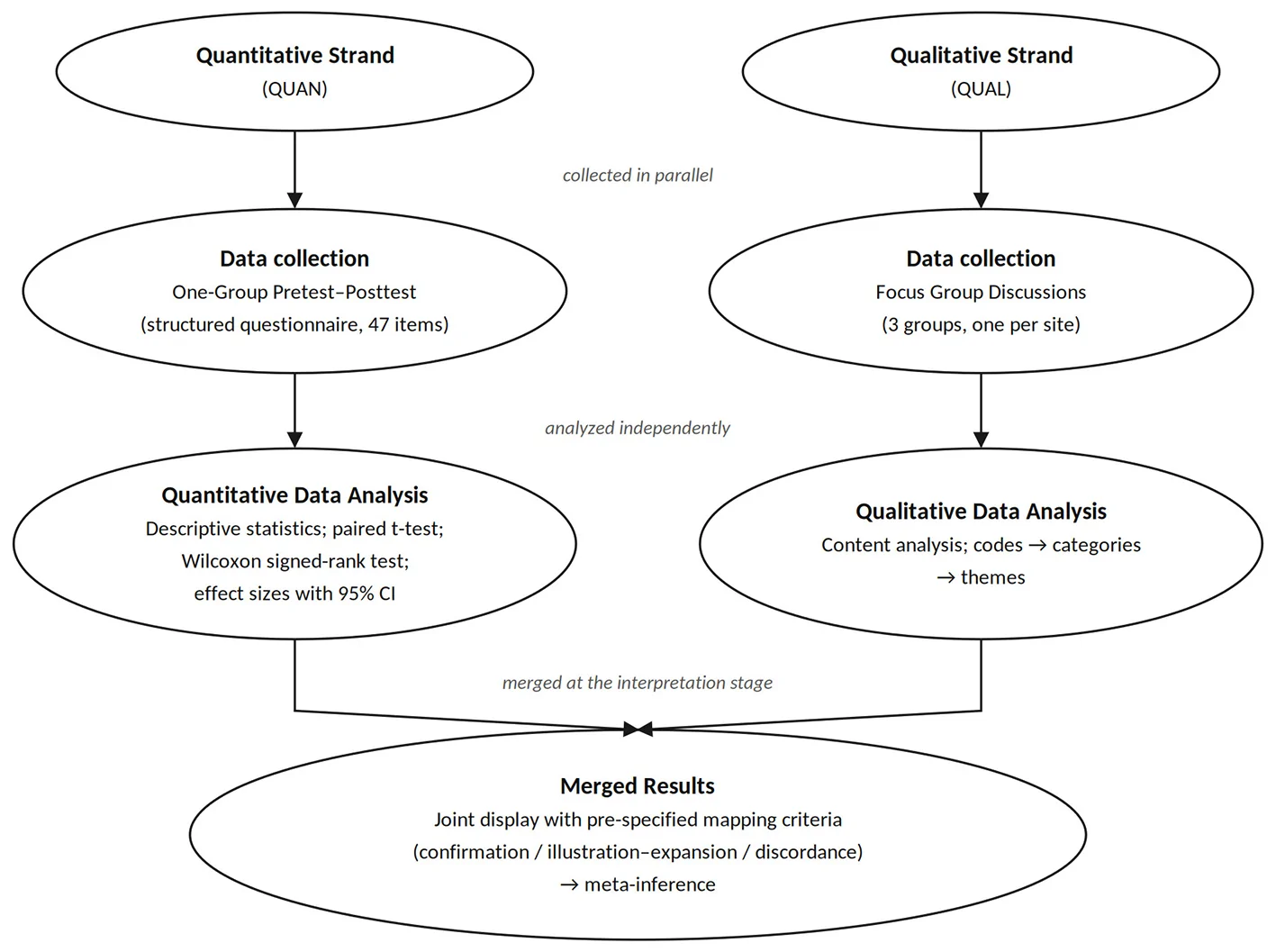
Flowchart of the convergent parallel mixed-methods design. The quantitative strand (single-group pretest–posttest, structured questionnaire) and the qualitative strand (focus group discussions) were conducted in parallel and analyzed independently, then merged in a joint display with pre-specified mapping criteria and interpreted through an explicit meta-inference.

### Rigor and trustworthiness

Credibility was supported by member checking, independent double coding, and triangulation across the two strands and the three sites. Dependability was supported by an audit trail of raw transcripts, code books, and analytic memos. Confirmability was addressed through the reflexive procedures described above, and through independent administration of the questionnaires. Transferability is supported by the detailed description of setting, participants, and program content given above and in Table 1.

### Reflexivity and the researcher–participant relationship

The research team designed the program, facilitated all sessions, and also conducted the qualitative strand; the first author, who moderated all three focus groups, had taught every participant repeatedly over the academic year. This dual role establishes an asymmetric relationship between researchers and participants and creates a clear risk of social desirability and courtesy bias, which we regard as the most serious threat to the qualitative findings and, given the self-report outcome measure, as a plausible influence on the quantitative findings as well. Several procedures were used to reduce, though not to remove, that risk. Questionnaires were administered by research assistants with no role in program delivery, completed anonymously, and linked only by participant-generated codes. Participants were told in writing and verbally, at enrolment and again at each data collection point, that neither the facilitators nor their supervisors would see individual responses, that no individual or site-level results would be reported to their organizations, and that their participation and their responses would have no bearing on employment, performance appraisal or access to future training. No supervisor received individual data at any stage; participating organizations received only the aggregate findings reported here. At the start of each focus group the moderator stated explicitly that critical and negative accounts were as useful to the study as positive ones, invited participants to describe what had not worked, and used open, non-directive probes without evaluative feedback; two note-takers who were not the designated mentor for that site recorded non-verbal responses and instances of disagreement. Coding was carried out independently by two researchers, one of whom was not the designated mentor for the site being coded, and disagreements were resolved with a third researcher. This is only partial independence: because all three facilitators taught every session, no member of the research team was unknown to any participant.

Critical and neutral accounts were present in the transcripts and were retained in the analysis rather than set aside; they are reported in the Results, within Theme 4, rather than confined to this section. In summary, participants described academic dissemination as the step they had practiced least, and several expressed doubt about sustaining the work under routine conditions once facilitator support ended. We nevertheless judge that the overall balance of the transcripts is likely to be more positive than it would have been under independent moderation, and this is stated as a limitation rather than treated as resolved.

### Ethical considerations

The study was approved by the Human Research Ethics Committee of Sirindhorn College of Public Health, Yala Province, Thailand (approval number SCPHYLIRB-2566/227) and was conducted in accordance with the Declaration of Helsinki and applicable national regulations. Before enrollment, all participants were informed of the identity of the researchers, the objectives and benefits of the study, the data collection procedure, the anonymity of responses, the confidentiality of storage, the fact that findings would be reported in aggregate, and their right to decline or withdraw at any time without any effect on their employment. Written informed consent was obtained from every participant, including separate consent for audio recording. Data will be destroyed 6 months after publication of the results.

## Results

### Participant characteristics

All 30 recruited participants completed the program and both assessments (retention 100%). Most were women (83.3%), with a mean age of 42.3 years (SD = 12.5, range 23–59) and a mean of 18.9 years of work experience (SD = 13.3, range 1–39). Registered nurses (50.0%) and public health academic officers (46.7%) predominated. Only five participants (16.7%) had previously conducted research. Full characteristics are shown in Table 3. Attendance and retention are reported here as feasibility indicators of the delivery format, not as evidence of program effect: all 30 participants attended all five sessions and completed both assessments, in a sample that had committed in advance to do so.

**Table 3:** Characteristics of participants (*n* = 30).

Characteristic	*N*	%
Gender		
Male	5	16.7
Female	25	83.3
Age, years (mean 42.33, SD 12.50, range 23–59)		
Work experience, years (mean 18.89, SD 13.25, range 1–39)		
Education level		
Bachelor's degree	25	83.3
Master's degree	5	16.7
Position		
Registered nurse	15	50.0
Public health academic officer	14	46.7
Traditional Thai medicine practitioner	1	3.3
Previous research experience		
Yes	5	16.7
No	25	83.3

SD, standard deviation. Percentages may not total 100 because of rounding.

### Quantitative strand

Self-reported research competency was higher at posttest than at pretest, overall and in every domain (Table 4). The overall mean rose from 3.13 (SD = 0.27) to 4.27 (SD = 0.35) on the five-point scale, a within-participant mean difference of 1.14 (95% CI 0.98–1.30; t(29) = 14.136; *p* < 0.001; Cohen's dz = 2.58). The largest domain increases were in motivation for research development (mean difference 1.50, 95% CI 1.19–1.81; dz = 1.81) and appreciation of the value of research (mean difference 1.34, 95% CI 1.03–1.65; dz = 1.62). The two domains analyzed non-parametrically also increased, with large effect sizes (research-supportive traits, *Z* = 4.613, *r* = 0.60; attitude toward research, *Z* = 4.607, *r* = 0.59). The smallest, though still large, increase was in the ability to present academic work (mean difference 0.87, 95% CI 0.56–1.18; dz = 1.04). At posttest, every domain mean exceeded 4.0 except knowledge of research development (3.77), which had also started from the lowest baseline (2.84). These values describe within-participant change in self-reported competency over one academic year; because no comparison group was studied, the statistical significance of these changes does not establish that the program caused them.

**Table 4:** Self-reported research competency of public health personnel before and after the empowerment-based capacity development program (*n* = 30).

Competency domain	Pretest mean (SD)	Posttest mean (SD)	Mean difference (95% CI)	*t/Z*	*p*	Effect size
1. Knowledge of research development	2.84 (0.51)	3.77 (0.64)	0.93 (0.63–1.23)	6.286^a^	< 0.001	dz = 1.15
2. Ability to conduct research	3.06 (0.51)	4.20 (0.49)	1.14 (0.91–1.37)	9.922^a^	< 0.001	dz = 1.81
3. Research-supportive personal traits	3.17 (0.44)	4.41 (0.41)	1.24	4.613^b^	< 0.001	r = 0.60
4. Attitude toward research development	3.08 (0.46)	4.40 (0.54)	1.32	4.607^b^	< 0.001	r = 0.59
5. Motivation for research development	2.98 (0.51)	4.48 (0.50)	1.50 (1.19–1.81)	9.924^a^	< 0.001	dz = 1.81
6. Appreciation of the value of research	3.19 (0.71)	4.53 (0.49)	1.34 (1.03–1.65)	8.863^a^	< 0.001	dz = 1.62
7. Application of findings to work improvement	3.44 (0.50)	4.37 (0.47)	0.93 (0.70–1.16)	8.146^a^	< 0.001	dz = 1.49
8. Ability to present academic work	3.39 (0.55)	4.26 (0.57)	0.87 (0.56–1.18)	5.712^a^	< 0.001	dz = 1.04
Overall	3.13 (0.27)	4.27 (0.35)	1.14 (0.98–1.30)	14.136^a^	< 0.001	dz = 2.58

^a^Paired t-test, df = 29; ^b^Wilcoxon signed-rank test. All tests one-tailed. CI, confidence interval; SD, standard deviation; dz, Cohen's d for paired samples; r = Z/√N. Confidence intervals are not reported for domains analyzed with the Wilcoxon test.

### Qualitative strand

Content analysis of the three focus groups, all conducted after the program, produced nine categories describing how participants retrospectively accounted for the change in their competency. The nine categories were grouped into four themes: crossing the expert barrier (Theme 1); from first publication to research as ordinary work (Theme 2); learning with others and putting findings to use (Theme 3); and the limits of intention — workload, dissemination and dependence on scaffolding (Theme 4). The first three themes describe what participants reported gaining; the fourth describes where that gain stopped, and is reported here rather than in the discussion of limitations because it is part of the phenomenon and not only a caveat about it. Categories are presented with illustrative quotations; participants are identified by number only.

#### Theme 1: crossing the expert barrier

The first theme describes the transition participants made from treating research as a specialist activity belonging to academics to treating it as a procedure they could execute. 25 of the 30 participants had never conducted research before the program, and their accounts of the early sessions are consistently accounts of exclusion: research was something read about, cited in policy documents, or supervised by outsiders, but not produced locally. What changed, in their telling, was not primarily the acquisition of facts about methodology but the discovery that the steps were finite, ordered and learnable — that a research process has a beginning and an end and can be followed. Four categories make up this theme: acquiring research knowledge, thinking systematically, searching for evidence, and gaining confidence in data analysis, the last of which participants described as the point at which the barrier finally fell, because statistics had been the element they most expected to be beyond them.

##### Acquiring research knowledge

Participants described moving from no working knowledge of research to an understanding of the research process as a sequence they could follow.

“*From the beginning of learning, I started from zero, but now, in phase 4 of the project, I have gained significant experience and research knowledge.” (Participant 2)*

“*I now understand the research process and steps, which I previously knew nothing about. I have also learned techniques and methods to gather information.” (Participant 19)*

##### Thinking systematically

Participants reported applying the research process to everyday service problems rather than only to the study they had completed.

“*With guidance from the instructors, I have developed a more structured and systematic way of thinking.” (Participant 2)*

“*One key takeaway was learning to systematically analyze and study problems encountered in the workplace using research methodologies.” (Participant 3)*

##### Searching for evidence

Access to and use of academic databases, particularly Thai Journals Online, was repeatedly described as a new and practical skill.

“*This was my first time conducting a full-scale research project, and I learned how to search for information effectively using ThaiJo.” (Participant 3)*

##### Gaining confidence in data analysis

Statistical analysis shifted from being an obstacle to being manageable.

“*After attending the training, I now understand statistics and data analysis better. I used to think it was difficult, but now it seems much easier.” (Participant 8)*

#### Theme 2: from first publication to research as ordinary work

The second theme describes what happened after participants completed a first piece of work. Completion, and for some participants publication, functioned less as an end point than as a reinterpretation of the work they were already doing: routine service activity that had previously looked like a burden separate from research came to look like research material. This reframing is the change participants themselves described as the most consequential, and it is the mechanism they invoked when explaining why they expected to continue. It also carries a caution that becomes visible in Theme 4: the reframing concerns the accessibility of research, not the ease of publishing it, and participants who described research as “simple” were describing the design and data collection stages rather than dissemination.

##### Publishing for the first time

Several participants completed and disseminated their first academic work during the program.

“*This was my first time completing a research project and getting it published.” (Participant 5)*

“*I never thought I would get my research published in my lifetime.” (Participant 6)*

##### Perceiving research as feasible

Participants reframed research from an additional burden to a way of examining work they were already doing.

“*Every task can be researched. This training made research feel simple.” (Participant 7)*

“*There's no need to stress over it — just take our regular work and develop it into a research study.” (Participant 21)*

#### Theme 3: learning with others and putting findings to use

The third theme describes the collective and applied dimensions of the change. Participants attributed a substantial part of their progress to working alongside colleagues from other professions and other sites — an arrangement that was a practical consequence of rotating the sessions across three settings rather than a designed intervention component — and to the requirement that each session end in a public presentation of progress. Two effects were described. The first was normative: seeing peers from comparable positions complete each stage made completion appear expected rather than exceptional. The second was practical: participants borrowed instruments, sampling strategies and analytic solutions from one another. Participants then linked their own findings to specific service or planning decisions, which is the point at which the exercise stopped being an academic requirement in their accounts.

##### Learning across professions

Working alongside colleagues from other disciplines and other sites was valued as a source of ideas and encouragement.

“*I met colleagues from different disciplines, exchanged research ideas, and learned from each other. It made public health work more enjoyable.” (Participant 7)*

##### Applying findings to practice

Participants linked their findings to concrete service or planning decisions.

“*This research helped clarify and analyze my work more academically, making it more applicable to my job.” (Participant 10)*

“*Next year, we plan to use our research findings to develop a project within our organization.” (Participant 14)*

#### Theme 4: the limits of intention — workload, dissemination and dependence on scaffolding

The fourth theme qualifies the first three, and is essential to a realistic reading of the quantitative results. Participants expressed confidence about initiating further studies, but that confidence was bounded in three consistent ways, and the boundaries correspond precisely to the domains in which the measured increase was smallest. First, service work took priority whenever the two competed, so project work advanced in the intervals that routine duties left rather than on a schedule. Second, dissemination — submission, revision and response to peer review — was the step participants had practiced least and the one they were least willing to attempt alone. Third, the confidence that participants did report was frequently expressed as confidence with the mentor available, not confidence independent of the program. These accounts are not incidental caveats: they identify the structural conditions under which the reported gains were achieved and are the participants' own explanation for why the technical and dissemination domains lagged behind the motivational ones.

##### Intending to continue

Participants expressed confidence that they could initiate further studies without external facilitation.

“*After completing this project, I feel confident that I can conduct research within my workplace in the future.” (Participant 12)*

##### Workload, dissemination and dependence on the mentor

*Participants described project work as competing directly with service delivery rather than being accommodated alongside it, and described the competition as routinely resolved in favor of service*.

“*The manuscript is still not finished, because when I am back at the health-promoting hospital the service work always comes first — the research waits until there is a quiet week, and there rarely is one.” (Participant 12)*

*Confidence about the earlier stages of the research process coexisted with explicit doubt about carrying work through to publication without continuing support, indicating that the scaffolding provided by the program had not yet been internalized at the dissemination stage*.

“*I can collect data and analyze it now, but I am not sure I could take a paper through to publication on my own; without the mentor reading my drafts I would probably stop at the report stage.” (Participant 7)*

A third pattern concerned projects that stalled between sessions rather than at any particular technical step, and participants attributed the stalling to events in the service rather than to the difficulty of the research.

“*Between session three and session four I did not open the file even once. There was a dengue outbreak in my area and everything else stopped. When I came back I had forgotten where I was, and I had to ask the mentor to explain my own analysis plan back to me.” (Participant 19)*

Others described a gap between what the program made thinkable and what their workplace permitted, which is a different limitation from either time or skill.

“*The training told us every task can be researched, and that is true. But nobody told my supervisor that. My name is on the same duty roster as before, so the research happens after hours or it does not happen.” (Participant 23)*

Taken together, these accounts indicate that the program produced conditional rather than autonomous research capability within its timeframe: participants had acquired a method and a reason to use it, but not yet the protected time or the independent editorial confidence that sustained research activity requires.

#### Variation across the three settings

Because the three sites were selected to represent district administration, hospital and primary care settings, we examined whether accounts differed by setting. The categories themselves appeared in all three groups, and no category was unique to one site; the differences were of emphasis and of constraint rather than of kind. Protecting time was described as hardest at the hospital site, where participants worked rotating shifts and reported that the two program days were recovered from days off rather than from duty time; the accounts of stalled projects quoted above both came from this group. Participants at the district public health office linked findings to service or planning decisions most readily, which they attributed to holding planning responsibility for their catchment area: three of the 10 described taking their results into the annual district health plan. Interprofessional exchange was valued most explicitly at the primary healthcare unit, the smallest of the three teams, where participants said the rotation gave them access to colleagues they would not otherwise meet. One account ran against these expectations: two hospital participants described the shift system as an advantage rather than a constraint, because a quiet night shift gave uninterrupted time for reading and writing that office-based colleagues did not have. These comparisons are descriptive and involve ten participants per site, so they indicate where setting may matter for implementation rather than establishing that it does.

### Integration of the two strands

The joint display (Table 2) applies the mapping criteria described in the Methods. Construct-level consistency (confirmation) between the strands was found for five of the eight domains: knowledge of research development, ability to conduct research, attitude toward research development, application of findings to work improvement, and ability to present academic work. For three domains the relationship was classified as illustration/expansion rather than confirmation, because the qualitative category elaborated a related but non-equivalent construct: research-supportive personal traits (mapped to “intending to continue”, which describes intention and self-efficacy rather than the trait items themselves), motivation for research development (mapped to “publishing for the first time”, which is an experience participants described as motivating rather than a measure of motivation), and appreciation of the value of research (mapped to “applying findings to practice”, which describes use rather than valuation). Two participant-reported outcomes were not represented anywhere in the 47-item instrument: interprofessional and cross-site exchange, and the reframing of research as part of routine service work rather than as an additional task. The second of these is one facet of the category “perceiving research as feasible”, whose other, attitudinal facet is mapped to the attitude domain; the two facets are separated in Table 2 rather than allowing one category to count both as matching and as not matching the instrument. No discordance was identified. The domain with the smallest quantitative increase, ability to present academic work, was also the one for which qualitative accounts were least detailed, with participants describing dissemination as the step they had practiced least.

### Meta-inference from the integrated findings

Bringing the two strands together supports one inference that neither strand supports on its own, and the qualitative strand supplies the explanation that the quantitative pattern alone would leave open. The ordering of measured change and the ordering of participants' accounts correspond: what changed most, in both strands, was the perceived value and ownership of research rather than technical and dissemination capability. Quantitatively, the four largest mean increases were all in dispositional domains motivation for research development (+1.50), appreciation of the value of research (+1.34), attitude toward research development (+1.32) and research-supportive personal traits (+1.24) whereas the three smallest were technical or applied: knowledge of research development and application of findings to work improvement (both +0.93) and ability to present academic work (+0.87). Knowledge also retained the lowest posttest mean (3.77). Standardized effect sizes qualify this ordering rather than contradict it: dz reflects the consistency of the difference scores as well as their size, and ability to conduct research reached the same dz as motivation (1.81) from a smaller mean increase (+1.14) because its difference scores varied little across participants.

The integrated inference concerns why that lag occurred, and here the qualitative strand does more than illustrate the numbers. A purely quantitative reading would attribute the lag to a theoretical sequence that empowerment acts on perceived control and value before technical mastery, which then accrues with practice. Theme 4 indicates that the sequence is at least as much structural as developmental. Participants did not describe technical and dissemination competencies as merely slower to develop; they described them as competing with service delivery for time that was not protected between sessions (Participant 12), and as requiring editorial scaffolding they did not expect to have once the program ended (Participant 7). On this reading, the smallest measured increase ability to present academic work is not evidence that dissemination is intrinsically harder to learn, but evidence that it was the stage least rehearsed within the program and the stage most exposed to time-poverty afterwards. The practical implication is different from, and more actionable than, the theoretical one: the domains that lagged would be addressed by protected writing time and by extending mentorship through submission and revision, rather than by adding methodological content. This is the kind of statement that neither strand yields alone the quantitative strand locates the lag, the qualitative strand identifies what produces it and it is the principal value of the integration for service administrators deciding how to fund programs of this type.

The two outcomes absent from the instrument add a further integrated observation: interprofessional exchange and the normalization of research within routine work were described by participants as central to the change, yet the 47-item scale contains no item for either, which suggests that the measurement model in this field does not fully capture the outcomes participants consider most consequential. Three qualifications bound this meta-inference and are stated here rather than only in the Limitations. First, it concerns participants' perceptions and self-assessments, at Kirkpatrick levels 1 and 2; it says nothing about demonstrated competence, workplace behavior, or research output. Second, it is not a causal statement: with a single-group design, maturation, testing effects, and the initial motivation of a self-selected group could each contribute to both the measured change and the accounts given. Third, the qualitative accounts were collected after the program and are retrospective reconstructions, susceptible to the same expectancy that may have inflated posttest scores; their value lies in explaining how participants understood the change, not in establishing that it occurred.

## Discussion

Public health personnel who completed the Empowerment-Based Capacity Development Program reported higher research competency at the end of the program than at the beginning, overall and in all eight domains, with a very large within-participant effect for the overall score (dz = 2.58) and large effects for each domain. These are changes in self-reported competency, corresponding to Kirkpatrick levels 1 and 2, and they are not evidence of demonstrated competence or of a causal program effect. Focus group accounts were consistent with the measured pattern and specified the mechanism participants themselves perceived: a shift from seeing research as an external, expert activity to seeing it as an extension of their own work. The integration in Table 2 shows that this consistency held at construct level for five domains, that three further domains were illustrated rather than confirmed, and that two dimensions described as central by participants interprofessional exchange and the normalization of research within routine practice were not represented in the questionnaire at all.

These findings are consistent with previous empowerment-based capacity building in Thai education and health settings. Improvements in research and development competency after empowerment and self-efficacy enhancement have been reported among secondary school teachers (22), and a program for health personnel in Phra Nakhon Si Ayutthaya province enabled participants to design and complete projects addressing local service problems (23). The parent model on which the present program was based reported comparable gains among village health volunteers and health officials (14). Our contribution is to document a similar pattern among district-level personnel with full service responsibilities, and to report the experiential account alongside the measured change; it is not to establish that the pattern is attributable to the program.

### Positioning relative to established research capacity building programs

Set against established research capacity building programs in low- and middle-income countries, the program evaluated here is modest, and we do not present it as an advance on them. The Rwandan Intermediate Operational Research Training course delivered a full year of training with intensive individual mentorship and reported 28 co-first-authored manuscripts, 27 of them published, from 53 trainees, with per-trainee and per-project costs documented (16). The Population Health Implementation and Training partnerships sustained research capacity building across five African countries for 5 years, including research administration, infrastructure, scholarships and 116 manuscripts (17). The Tanzanian Community of Young Research Peers built a durable structure combining vertical and horizontal mentorship for early-career researchers (18). All three reported objective research outputs; the present study reported none. Some participants described completing and publishing their first academic work, but no protocol, project or publication was verified, counted or followed up by the research team, so those accounts cannot serve as output data. This is a substantive difference in the strength of evidence, not only in scale. What this evaluation adds is evidence about a different and common constraint: whether an abbreviated, ten-day, mentorship-supported research cycle can be delivered within one academic year to district-level personnel who remain in full service roles, how acceptable such personnel find it, and how they account for what changes. The complete retention and the internal coherence of participants' accounts suggest the format is feasible and acceptable in favorable settings. Whether it yields research outputs approaching those of the more intensive programs above is unknown from these data and is the most important next question; the models cited here also indicate what a stronger evaluation would report, namely completion, publication and cost per trainee.

The pattern of domain-level results is informative. Motivation and appreciation of research changed most, whereas knowledge showed the lowest posttest mean and dissemination the smallest increase. Empowerment theory predicts this ordering the process works first on perceived control and value, and only then on technical mastery, which continues to develop with repeated practice (12, 13) but our qualitative data indicate that the theoretical sequence is not the whole explanation. Participants located the lag in conditions rather than in stages: service work took precedence over project work between sessions, and dissemination was both the least rehearsed activity within the program and the one participant were least prepared to attempt without an available mentor. The ordering is therefore consistent with empowerment theory and with the Tanzanian evidence that motivation, professional growth, and collaboration are the dominant enablers of research participation among health providers (8), and with the meta-narrative conclusion that capacity strengthening delivered as technique alone rarely persists (10); but the actionable reading is that the lagging domains reflect unprotected time and a truncated mentorship window rather than an unmet need for further methodological content. The practical implication is that programs of this kind should not be evaluated by knowledge tests alone, and should not be extended by adding lectures; the corresponding methodological implication is that they should not be evaluated by self-report alone either.

The specific skill gains reported by participants literature searching, evidence appraisal, data synthesis, and statistical interpretation correspond to the evidence-based information needs documented for the public health workforce (32) and to competency frameworks developed for community health personnel in China (33, 34) and infection prevention practitioners in Korea (35). Participants' stated intention to continue, and their plans to apply their findings within their organizations, are consistent with the concept of the learning organization, in which individual learning is retained and reused at the organizational level (36). Whether that retention actually occurred cannot be established from the present data, which contain no organizational-level measurement and no follow-up beyond 2 weeks.

### Methodological contribution

This study also illustrates the practical value, and the limits, of a convergent parallel design in evaluating capacity-building programs. Quantitative measurement established the magnitude of self-reported change across a defined competency construct, while the qualitative strand identified the processes through which participants believed the change occurred and revealed two outcomes outside the measurement model. Presenting the merger as a joint display with pre-specified mapping criteria and an explicit fit classification (31) makes the inferential steps visible and contestable by readers, which is uncommon in this literature (21); applying those criteria conservatively also reduced the number of domains we could describe as confirmed to five of the eight, where the looser thematic reading taken in the submitted version would have described all eight as confirmed; we regard the conservative count as the more defensible one. The limits are equally clear: because both strands rest on participant self-report and were collected by the team that delivered the program, convergence between them is not independent corroboration, and shared bias is a plausible explanation for part of it. The two expansion findings nevertheless provide an empirically grounded basis for revising the competency instrument.

### Implications for policy and practice

Three implications follow, offered as hypotheses for stronger designs to test rather than as established recommendations. First, capacity development for frontline public health personnel may be more workable when embedded in routine work and extended over time than when delivered as a single course; participants attributed more value to the repeated cycle of writing, presenting, and feedback than to lecture content. Second, organizational sponsorship appears to matter: sites were selected partly on the written commitment of the head of the organization to release staff, and participants attributed their persistence to that protection — which also means these findings may not transfer to organizations unable or unwilling to provide it. Third, because dissemination showed the smallest increase and was the least rehearsed activity, programs of this type should extend beyond a first draft to structured support for submission, revision, and response to peer review.

### Limitations

Several limitations qualify these findings, and together they define what the study can and cannot claim. The design was a single group measured before and after the program, with no comparison group; maturation, history, testing and re-testing effects, regression to the mean, and concurrent organizational initiatives cannot be excluded, and no causal inference about the program is warranted. Both the organizations and the individuals were self-selected and highly selected: organizations volunteered and had to demonstrate readiness, and participants committed in writing in advance, so the results are likely to overstate what the same program would achieve in routine conditions with unselected staff. The outcome measure was entirely self-reported, and no objective indicator of research competence or productivity protocols submitted for ethical review, completed projects, or peer-reviewed publications was collected; the study therefore reaches Kirkpatrick levels 1 and 2 only. The instrument carried validity evidence from a prior study and a small pilot; no construct-validity evidence was generated in the present sample, which was too small for factor analysis, so the domain scores should be read as descriptive subscales. Because the research team both delivered the program and moderated the focus groups, social desirability and courtesy bias are plausible in both strands despite the safeguards described in the Methods, and convergence between two self-reported strands collected under the same conditions is weaker evidence than convergence between independent sources. The qualitative data were collected only after the program, so change was reconstructed retrospectively rather than observed. The posttest was administered within 2 weeks of the final session, so retention and sustained application are unknown. Finally, the study was conducted in three purposively selected Thai public health settings, and its findings are offered as transferable insight rather than as generalizable estimates.

### Future research

Controlled designs ideally cluster-randomized or stepped-wedge across districts, which would also address the ethical objection to withholding the program from individuals, since whole sites receive it in sequence with follow-up between 6 and 12 months are needed to establish effect and durability. Future evaluations should complement self-report with objective indicators at Kirkpatrick levels 3 and 4, including protocols submitted for ethical review, completed projects, peer-reviewed publications, and documented changes in local service delivery, and should report completion and cost per trainee so that formats can be compared with established programs (16). A distinct instrument-development agenda also follows directly from the expansion findings. The two outcomes participants described as central interprofessional and cross-site exchange, and the reframing of research as part of routine service work rather than as an additional task have no corresponding item in the 47-item scale used here, and, to our knowledge, no equivalent item in the competency instruments developed for community health personnel in China (33, 34) or for infection prevention practitioners in Korea (35). We therefore call explicitly for revision of research competency scales for practitioner populations, adding a collaboration and knowledge-exchange dimension and a dimension capturing the integration of research into routine work, and for psychometric evaluation of the revised instrument in adequately powered samples, including confirmatory factor analysis. Until such items exist, evaluations of practitioner research capacity will continue to omit outcomes that the practitioners themselves consider most important. The model should also be tested in multidisciplinary teams and in settings with weaker mentorship infrastructure, and, given Theme 4, trials should test protected writing time and mentorship extended through submission and revision as explicit program components.

## Conclusion

Among 30 self-selected Thai public health personnel, completion of an empowerment-based, practice-embedded capacity development program was associated with large within-participant increases in self-reported research competency across all eight measured domains, and participants' retrospective accounts explained those increases in terms of greater control, confidence, and integration of research into routine work. Complete attendance and retention, together with participants' accounts of the program as workable alongside routine service duties, indicate that the format is feasible and acceptable in settings with organizational support and merits more rigorous evaluation; the consistency between the two strands, which are both self-reported and therefore not independent of one another, lends coherence to that reading rather than corroboration of it. Two outcomes the measurement model did not capture — interprofessional exchange and the normalization of research within routine work — also suggest that the instrument used in this field needs revision. Because the design was single-group, the sample small and self-selected, and both strands self-reported at Kirkpatrick levels 1 and 2, these findings should be read as preliminary feasibility and implementation evidence and not as evidence that the program caused the observed change. Controlled evaluations with longer follow-up and objective research-productivity indicators are required before wider scale-up.

## Data Availability

The original contributions presented in the study are included in the article/Supplementary material, further inquiries can be directed to the corresponding author.
