# Supplementary material for: Changes in research competency of public health personnel following an empowerment-based capacity development program in Thailand: a convergent parallel mixed-methods study
Source: Front Public Health. 2026 Sep 23;14:1950760. doi: 10.3389/fpubh.2026.1950760 (PMC13642790; doi:10.3389/fpubh.2026.1950760)
Supplement: Supplementary file 1 [file Table_1.docx]

Supplementary Material

*Manuscript: Empowerment-Based Capacity Development Improves the Research Competency of Public Health Personnel in Thailand: A Convergent Parallel Mixed-Methods Study*

# 1 1 Supplementary Table 1 | GRAMMS checklist

Good Reporting of A Mixed Methods Study (GRAMMS; O’Cathain, Murphy and Nicholl, 2008). Section numbers refer to the submitted manuscript.

| **#** | **GRAMMS item** | **Where addressed in the manuscript** |
| --- | --- | --- |
| 1 | Describe the justification for using a mixed methods approach to the research question | Introduction, final two paragraphs (research gap and rationale for combining measured change with participant accounts) |
| 2 | Describe the design in terms of purpose, priority and sequence of methods | Section 2.1 (convergent parallel design; equal priority QUAN + QUAL; concurrent data collection); Figure 2 |
| 3 | Describe each method in terms of sampling, data collection and analysis | Sections 2.2–2.4 and 2.7 (quantitative); Sections 2.6 and 2.8 (qualitative); Section 2.9 (analysis of both strands) |
| 4 | Describe where integration has occurred, how it has occurred and who has participated in it | Section 2.10 (integration at the interpretation stage using a joint display); Section 3.4 and Table 4; integration performed jointly by all authors |
| 5 | Describe any limitation of one method associated with the presence of the other method | Section 4.3 (qualitative data collected only after the intervention by the same team that delivered it; potential social desirability affecting both strands) |
| 6 | Describe any insights gained from mixing or integrating methods | Sections 3.4 and 4.1 (two expansion findings not captured by the 47-item instrument; implications for revising the measure) |

Reference: O’Cathain A, Murphy E, Nicholl J. The quality of mixed methods studies in health services research. J Health Serv Res Policy (2008) 13:92–8.

# 2 2 Supplementary Table 2 | COREQ checklist

Consolidated Criteria for Reporting Qualitative Research (COREQ; Tong, Sainsbury and Craig, 2007), applied to the qualitative strand. Items marked [confirm] must be completed by the authors before submission.

| **COREQ item** | **Location** | **Note** |
| --- | --- | --- |
| Domain 1: Research team and reflexivity |  |  |
| 1. Interviewer/facilitator | Section 2.8 | Focus groups moderated by the first author, supported by two trained note-takers |
| 2. Credentials | Section 2.5 | [confirm] e.g., PhD, RN |
| 3. Occupation | Section 2.5 | Public health researchers / academic staff |
| 4. Gender | Not reported | [confirm] state gender of the moderator |
| 5. Experience and training | Section 2.5 | Facilitators had prior experience mentoring practitioner research |
| 6. Relationship established | Section 2.11 | Yes — the research team also delivered the intervention; addressed reflexively |
| 7. Participant knowledge of the interviewer | Section 2.12 | Participants were informed of the researchers’ identity and study objectives at consent |
| 8. Interviewer characteristics | Section 2.11 | Reflexive statement on the dual role of trainer and researcher |
| Domain 2: Study design |  |  |
| 9. Methodological orientation and theory | Sections 2.1 and 2.9 | Convergent parallel mixed-methods design; content analysis (Miller and Crabtree) |
| 10. Sampling | Section 2.3 | Purposive sampling with stated inclusion criteria |
| 11. Method of approach | Section 2.2 | Open call to organizations; face-to-face recruitment |
| 12. Sample size | Section 2.3 | 30 participants; three focus groups of 10 |
| 13. Non-participation | Section 2.3 | No withdrawals; 100% retention |
| 14. Setting of data collection | Section 2.8 | At each of the three participating organizations |
| 15. Presence of non-participants | Section 2.8 | Only participants, the moderator, and two note-takers were present |
| 16. Description of sample | Section 3.1 and Table 2 | Reported |
| 17. Interview guide | Section 2.6 | Semi-structured guide with one broad opening question and non-directive probes; expert-reviewed; provided as Supplementary Material 4 |
| 18. Repeat interviews | Section 2.8 | No repeat focus groups were held |
| 19. Audio/visual recording | Section 2.8 | Audio recorded with written permission; verbatim transcription |
| 20. Field notes | Section 2.8 | Taken by two note-takers during each session |
| 21. Duration | Section 2.8 | 90–120 minutes per focus group |
| 22. Data saturation | Not reported | [confirm] state whether saturation was discussed or reached |
| 23. Transcripts returned | Section 2.8 | Moderator summary verified by participants (member checking); [confirm] whether full transcripts were returned |
| Domain 3: Analysis and findings |  |  |
| 24. Number of data coders | Section 2.9 | Two independent coders; third researcher resolved disagreements |
| 25. Description of the coding tree | Section 2.9 / Supplementary Material | [confirm] attach the code book if available |
| 26. Derivation of themes | Section 2.9 | Derived inductively from the data |
| 27. Software | Not reported | [confirm] state software used, or that coding was manual |
| 28. Participant checking | Section 2.8 | Member checking at the end of each focus group |
| 29. Quotations presented | Section 3.3 | Quotations presented and identified by participant number |
| 30. Data and findings consistent | Section 3.3 | Each category supported by at least one quotation |
| 31. Clarity of major themes | Sections 3.3 and 3.4 | Nine categories presented and integrated |
| 32. Clarity of minor themes | Section 3.3 | Categories with fewer supporting accounts identified (e.g., 3.3.3, 3.3.4, 3.3.9) |

Reference: Tong A, Sainsbury P, Craig J. Consolidated criteria for reporting qualitative research (COREQ). Int J Qual Health Care (2007) 19:349–57.

# 3 3 Supplementary Material 3 | Research competency questionnaire (English translation)

The instrument was adapted from Semrum et al. (2018) and administered in Thai. Part 1 records seven participant characteristics. Part 2 contains 47 items rated from 1 (strongly disagree / least frequent practice) to 5 (strongly agree / most frequent practice). The structure is given below; the authors should paste the full English translation of each item into the right-hand column before submission, and retain the original Thai version as an additional file if requested by the editor.

| **Part / domain** | **Items** | **Item wording (English translation)** |
| --- | --- | --- |
| Part 1. Participant characteristics | 7 | Gender; age; education level; position; years of work experience; previous research experience; previous research training |
| Part 2.1 Knowledge of research development | 7 | [insert items 1–7] |
| Part 2.2 Ability to conduct research | 9 | [insert items 8–16] |
| Part 2.3 Research-supportive personal traits | 7 | [insert items 17–23] |
| Part 2.4 Attitude toward research development | 4 | [insert items 24–27] |
| Part 2.5 Motivation for research development | 4 | [insert items 28–31] |
| Part 2.6 Appreciation of the value of research | 4 | [insert items 32–35] |
| Part 2.7 Application of findings to work improvement | 6 | [insert items 36–41] |
| Part 2.8 Ability to present academic work | 5 | [insert items 42–46] |
| Total (Part 2) | 47 | Item-objective congruence 0.67–1.00; Cronbach’s α = 0.84 |

Note: domain item counts sum to 46 in the manuscript text as currently written; the authors should reconcile the item allocation so that the eight domains total 47 items.

# 4 4 Supplementary Material 4 | Focus group discussion guide

The guide below reproduces the structure described in Section 2.6 and 2.8 of the manuscript. Authors should replace it with the verbatim guide actually used, translated into English.

**Opening (moderator script)**

Thank you for taking part. There are no right or wrong answers. We are equally interested in what did not work for you as in what did. Nothing you say will affect your work or your relationship with your organization, and no names will appear in any report. May we begin recording?

**Main question**

1. Since you joined this program, how has your ability to produce academic work from your own routine work changed — and what, if anything, has not changed?

**Non-directive probes**

• Can you give an example from your own work?

• What was the most difficult part of the process for you?

• What made it possible for you to keep going between sessions?

• What would have happened if the program had stopped after the second session?

• What, if anything, would you do differently now with a new problem in your area?

• What has changed, if anything, in how your colleagues or your organization respond to research?

• What did not work well, or what would you change about the program?

**Closing**

Summary of the main points by the moderator, followed by an invitation to correct or add to the summary (member checking).

# 5 5 Note on supplementary figures

Figure 1 (conceptual framework) and Figure 2 (convergent parallel design flowchart) are submitted as separate figure files with the main manuscript, not as supplementary material. Supplementary images, if added, must be 300 dpi and in RGB color mode.
